# Risk Factors of Pneumothorax after Endobronchial Ultrasound-Guided Transbronchial Biopsy for Peripheral Lung Lesions

**DOI:** 10.1371/journal.pone.0049125

**Published:** 2012-11-07

**Authors:** Chun-Ta Huang, Sheng-Yuan Ruan, Wei-Yu Liao, Yao-Wen Kuo, Chi-Ying Lin, Yi-Ju Tsai, Chao-Chi Ho, Chong-Jen Yu

**Affiliations:** 1 Department of Internal Medicine, National Taiwan University Hospital, Taipei, Taiwan; 2 Department of Traumatology, National Taiwan University Hospital, Taipei, Taiwan; 3 Department of Internal Medicine, National Taiwan University Hospital, Yun-Lin Br., Yunlin, Taiwan; 4 School of Medicine, College of Medicine, Fu-Jen Catholic University, Taipei, Taiwan; 5 Graduate Institute of Epidemiology and Preventive Medicine, National Taiwan University, Taipei, Taiwan; NIH, United States of America

## Abstract

**Background:**

The risk of endobronchial ultrasound-guided transbronchial biopsy-related pneumothorax is a major concern and warrants further studies. The aim of our study was to estimate the risk of pneumothorax after this procedure and identify its risk factors.

**Methods:**

From 2007 to 2011, 399 patients who underwent endobronchial ultrasound-guided transbronchial biopsy for peripheral lung lesions were included in this study. The variables analyzed included patient factors, lesion factors and procedure factors. Multivariate logistic regression analysis was used to identify independent risk factors for pneumothorax.

**Results:**

The incidence of pneumothorax was 3.3% (13/399). Chest tube placement was required for 31% (4/13) of pneumothoraces. Independent risk factors for pneumothorax included pulmonary emphysema (OR, 55.09; 95% CI, 9.37–324.03; p<0.001) and probe position adjacent to the lesion (OR, 17.01; 95% CI, 2.85–101.64; p = 0.002). The number of biopsy specimens, age, sex, history of prior lung surgery and lesion size, location and character did not influence the risk of pneumothorax in our analyses.

**Conclusions:**

The risk of pneumothorax after endobronchial ultrasound-guided transbronchial biopsy is low. To further reduce the risk of pneumothorax, every effort should be made to advance the endobronchial ultrasound probe into the bronchus where it is imaged within the target lesion before embarking on transbronchial biopsy.

## Introduction

Since its advent, endobronchial ultrasound (EBUS) has been widely used to increase the diagnostic yield of transbronchial biopsy for peripheral lung lesions and has had a favourable safety profile. Although rare, complications do exist and pneumothorax and bleeding are the two most frequently encountered. [1]–[3] A recent meta-analysis demonstrated that the pooled rates of pneumothorax and intercostal catheter drainage of pneumothorax across 14 studies were 1.0% and 0.4%, respectively. [4] The patients’ risk of developing pneumothorax after EBUS-guided transbronchial lung biopsy (TBB) may be identified using risk factor analysis; however, it has not been systematically investigated, to our knowledge. Thus, the purpose of this study was to evaluate the risk factors for pneumothorax following EBUS-guided TBB in a large population. In particular, potentially modifiable risk factors associated with pneumothorax were sought in the hope of minimizing pneumothorax.

## Materials and Methods

### Study Subjects

From January 2007 to December 2011, all patients who underwent EBUS-guided TBB for peripheral lung lesions and had a post-procedure chest x-ray at the National Taiwan University Hospital were identified from our bronchoscopy registry and were the study subjects. Peripheral lung lesions were defined as lesions being surrounded by the lung parenchyma without evidence of endobronchial abnormalities. This study was approved by the Research Ethics Committee of the National Taiwan University Hospital and written informed consent was obtained from all patients before conducting the bronchoscopy procedure.

### Data Collection

**Figure 1:**
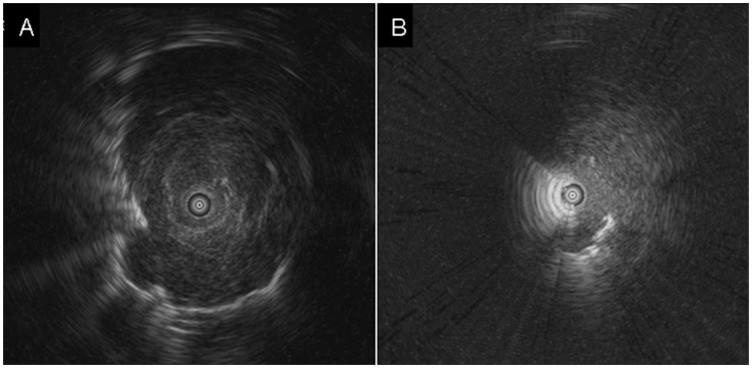
Endobronchial ultrasound images showing that the probe was located (A) within the lesion and (B) adjacent to the lesion.

Characteristics of the patients, lesions and procedures were collected to determine the risk factors for the occurrence of pneumothorax. The patient characteristics included the age, sex, presence of emphysematous change in the same lobe where the lesion was located on the CT scan, and history of surgery on the side of the lung where the biopsy was taken. The lesion characteristics were the size, lobar location and CT appearance of the lesion. The procedure characteristics included the number of biopsy specimens and position of the EBUS probe relative to the target lung lesion. All chest x-rays and CT scans were examined by two independent pulmonologists. The lesion size was measured as the largest diameter in the axial plane of the CT scan. The probe position was classified as either within (Figure 1A) or adjacent (Figure 1B) to the lesion, as previously described. [5] Pneumothorax was defined as the presence of air within the pleural space and was detected by the chest x-ray.

### Biopsy Procedures

Bronchoscopy was performed after local anaesthesia with 5 mL of 2% lidocaine sprayed or nebulised into the nasal, pharyngeal and laryngeal mucosa with or without intramuscular administration of meperidine 50 mg. Additionally, one or two aliquots of 1 mL of 2% lidocaine were instilled onto the larynx, carina and bronchial tree throughout the procedure. In 2007, an Olympus BF-1T240 video bronchoscope (Olympus, Tokyo, Japan) with a 2.6-mm working channel and an endoscopic ultrasound system (SSD-550, Aloka, Tokyo, Japan), equipped with a 20-MHz radial-type flexible probe were used. The settings of the ultrasound system were as follows: system gain 38 dB; time gain compensation 37; focal position 2 cm; imaging depth 4 cm. After 2008, an Olympus BF-P260F video bronchoscope (Olympus, Tokyo, Japan) with a 2.0-mm working channel was employed and EBUS was performed using an endoscopic ultrasound system (EU-M30S, Olympus, Tokyo, Japan), equipped with a 20-MHz radial-type flexible probe. The settings of the ultrasound system were as follows: system gain 9 dB; time gain compensation 4; imaging depth 4 cm. During the bronchoscopy, oxygen was delivered to the patient via a nasal cannula and continuous pulse oximetry was routinely used to monitor the patient’s blood oxygen saturation. EBUS-guided TBB was performed according to our previously described method. [6] The biopsy was repeated until at least three specimens were obtained.

### Statistical Analysis

Data were presented as mean±standard deviation or number (percent) as appropriate. The patients with or without pneumothorax after EBUS-guided TBB were compared. The variables evaluated were classified into patient factors (age, sex, pulmonary emphysema, history of lung surgery), lesion factors (size, location, CT appearance) and procedure factors (number of specimens, probe position, year of the procedure). Categorical variables were compared using the chi-square test or Fisher’s exact test and continuous variables were compared using the t test. The variables with a p value of less than 0.2 in the univariate analysis were entered into multivariate logistic regression analysis. A two-sided p value of less than 0.05 indicated statistical significance. The analyses were conducted using SPSS statistical software (SPSS version 15; SPSS; Chicago, IL).

## Results

A total of 399 consecutive patients (244 men and 155 women) were included in the study. The mean age was 64±13 years (range, 20 to 99 years). The average lesion size was 3.4±1.5 cm (range, 1.0 to 5.8 cm). Forty-five (11%) patients had emphysematous change in the same lobe of the lung lesion and 6 (1.5%) patients had received pulmonary surgery on the same side as the lesion. Table 1 summarizes the characteristics of the study cohort.

**Table 1:** Characteristics of patients included in the study*.

Variables	Total patients (n = 399)
Age, yr	64±13
Male sex	244 (61)
Prior lung surgery	6 (2)
Lesion size, cm	3.4±1.5
Lesion location	
Right upper lobe	114 (29)
Right middle lobe	40 (10)
Right lower lobe	72 (18)
Left upper lobe	103 (26)
Left lower lobe	70 (18)
Emphysema on CT scan	45 (11)
Character of the lesion	
Solid	339 (85)
Others#	60 (15)
Probe position	
Within	301 (75)
Adjacent to	98 (25)
No. of specimens	
3	191 (48)
4	61 (15)
5	55 (14)
6	49 (12)
≧7	43 (11)
Diagnosis	
Malignancy	220 (55)
Metastasis	14 (4)
Tuberculosis	16 (4)
Infection	10 (3)
Benign process	30 (8)
No diagnosis	109 (27)
Year of the procedure	
2007	60 (15)
2008–2011	339 (85)

* Data are presented as mean±SD or No. (%).

# Cavity, mixed ground-glass opacity, and pure ground-glass opacity.

Pneumothorax developed in 13 (3.3%) of the 399 patients. In one (7.7%) case, pneumothorax was completely evacuated with an intercostal catheter and manual aspiration and tube thoracostomy was indicated in 4 cases (31%). The remaining 8 patients required only observation and supplemental oxygen therapy. There was no mortality attributable to the development of pneumothorax.

From the univariate analyses, the factors significantly increasing the risk of pneumothorax were the presence of emphysema in the same lobe (p<0.001), non-solid lung lesion (p = 0.001) and EBUS probe positioned adjacent to the lesion (p = 0.004) (Table 2). The independent risk factors for pneumothorax in increasing order of p values obtained in a multivariate logistic regression model (Table 3) were pulmonary emphysema (OR, 55.09; 95% CI, 9.37–324.03; p<0.001) and probe adjacent to the lesion (OR, 17.01; 95% CI, 2.85–101.64; p = 0.002).

**Table 2:** Univariate analysis to determine risk factors for pneumothorax*.

Variables	Pneumothorax (n = 13)	No pneumothorax (n = 386)	p value
Age, yr			
	67±13	64±13	0.411
≧65	9 (61)	200 (52)	0.216
<65	4 (31)	186 (48)	
Sex			0.236
Male	10 (77)	234 (61)	
Female	3 (23)	152 (39)	
Prior lung surgery			0.181
Yes	1 (8)	5 (1)	
No	12 (92)	381 (99)	
Lesion size, cm			
	3.1±1.6	3.4±1.5	0.381
≧3	6 (46)	220 (57)	0.438
<3	7 (54)	166 (43)	
Lesion location			0.084
Upper lobes	10 (77)	203 (53)	
Middle and lower lobes	3 (23)	183 (47)	
Emphysema on CT scan			<0.001
Yes	9 (69)	36 (9)	
No	4 (31)	350 (91)	
Character of the lesion			0.001
Solid	6 (46)	333 (86)	
Others#	7 (54)	53 (14)	
Probe position			0.004
Within	5 (39)	296 (77)	
Adjacent to	8 (61)	90 (23)	
No. of specimens			
	3.9±1.1	4.2±1.4	0.327
≧4	6 (46)	202 (52)	0.661
<4	7 (54)	184 (48)	
Diagnosis			0.528
Diagnosis achieved	11 (85)	279 (72)	
No diagnosis	2 (15)	107 (28)	
Year of the procedure			0.999
2007	2 (15)	58 (15)	
2008–2011	11 (85)	328 (85)	

* Data are presented as mean±SD or No. (%).

# Cavity, mixed ground-glass opacity, and pure ground-glass opacity.

**Table 3:** Multivariate analysis to determine independent risk factors for pneumothorax.

Variables	Oddsratio	(95% confidenceinterval)	p value
Pulmonary emphysema	55.09	(9.37–324.03)	<0.001
Probe adjacent to the lesion	17.01	(2.85–101.64)	0.002
Non-solid lesion	3.98	(0.99–16.06)	0.052
Prior lung surgery	3.95	(0.31–49.79)	0.287
Lesion in upper lobes	3.34	(0.65–17.21)	0.149

## Discussion

The present study is dedicated to examine the risk factors for pneumothorax after EBUS-guided TBB in a large patient population. The pneumothorax rate was 3.3% and the presence of emphysema in the same lobe on the CT scan and position of the probe adjacent to the lung lesion were two recognized risk factors for pneumothorax. To further reduce the risk of pneumothorax, it is suggested that the EBUS probe should be introduced inside the target lesion if technically feasible.

EBUS-guided TBB has proven to be a useful and safe procedure for evaluation of a variety of lung diseases and the common and potentially catastrophic complication is pneumothorax. [1]–[4] After EBUS-guided TBB, the incidence of pneumothorax ranges from 0% to 5.1% and chest tube placement is required in approximately half of the total pneumothorax cases. [1]–[3], [5]–[10] Thus, the incidences of pneumothorax and tube thoracostomy for pneumothorax in our study were comparable to those of the aforementioned studies. Fortunately, no deaths due to pneumothorax were reported in our and prior studies.

Emphysema is pathologically characterized as destruction of alveolar architecture, with irreversible enlargement of airspaces distal to the terminal bronchioles. [11] The CT findings of emphysema are focal low attenuation areas, usually without visible walls. [12] In the present study, we found that if emphysema was detected in the same lobe of the target lesion, the risk of postbiopsy pneumothorax remarkably increased (OR, 55.09; 95% CI, 9.37–324.03). The anatomical derangement of emphysema may weaken the lung structure and predispose lung parenchyma to any kind of insults (i.e., lung biopsy here). Also, the disruption of dilated airspaces and lack of elastic recoil may prevent rapid sealing of the air leak. [13] Thus, it is anticipated that the distribution of emphysema is the most important effect of the disease process on the risk of developing clinically evident pneumothorax after EBUS-guided TBB. Indeed, the increased risk does not preclude such a procedure but helps us to be prepared ourselves and patients for potential pneumothorax.

Placing the EBUS probe within the lung lesion has been proved a significant predictor of higher diagnostic yield during EBUS-guided TBB. [2], [5], [6] Our study further identified probe position to be an important determinant of the development of pneumothorax. When the probe was advanced within the lesion on the EBUS image, the risk of pneumothorax was significantly decreased. The safety in terms of pneumothorax comes about because the bronchoscopists take biopsies when the lung lesion is well localized by EBUS. This means that the biopsy forceps will less likely touch the visceral pleura, the way pneumothorax develops. Thus, it is crucial to introduce the probe into the bronchus where it is imaged within the target lesion to increase the diagnostic yield as well as decrease the risk of pneumothorax.

In patients undergoing conventional TBB, the number of biopsy specimens obtained was a significant predictive factor for the development of pneumothorax. [14] However, our study investigating EBUS-guided TBB did not show such a relationship. Further, previous investigators did not find a correlation between the number of specimens retrieved and pneumothorax development in studies of conventional CT-guided and CT fluoroscopy-guided lung biopsy. [15]–[17] Regarding the lesion size, it correlated strongly with the development of pneumothorax in CT-guided biopsy of the lung but did not influence the risk of pneumothorax in EBUS-guided TBB as shown in this study. [18] As the lesion becomes smaller, it is more difficult for the biopsy needle to hit the lesion during CT-guided lung biopsy, making it more likely for specimens to be retrieved from the periphery of the lung lesion or the lung itself. As a result, pneumothorax may easily develop after such a procedure. On the contrary, we perform TBB after the lung lesion is precisely localized by EBUS and this will ensure that the specimens can be correctly obtained from the lesion. Thus, a small lesion size may not be identified as a risk factor for pneumothorax. Other factors investigated, namely, age, sex, history of prior lung surgery and lesion location and character had no predictive value in our analyses. Also, some of these factors have been proved to be independent risk factors for the development of pneumothorax after other lung biopsy procedures. [15], [19] This indicates that the risk factors for postbiopsy pneumothorax may be different for a variety of diagnostic modalities and reinforces the value of the present study. Another reason is that despite our large sample size, the relatively low number of pneumothoraces may well have masked an effect of one or more of these variables.

Our study has some limitations. First, whereas chest CT is superior to the chest x-ray in visualizing pneumothorax, [20] none of the patients in this study had a chest CT scan after the biopsy procedure. As a result, the true incidence of pneumothorax might be underestimated. Second, EBUS-guided TBB was performed by a cooperative team consisting of operators with varying degrees of experience in our institution, even though they were under supervision of more experienced specialist pulmonologists. In this regard, we could not evaluate the effect of operator expertise with the procedure on pneumothorax, yet it has been shown in many studies that the level of experience of the operators does not affect the risks of pneumothorax. [14], [16], [21] Finally, multiple testing for multiple variables, as conducted here, is surely associated with an inflated alpha error. Despite this concern, variables with a high odds ratio may represent true risk factors and attention should be paid to the patients with such variables.

In summary, EBUS-guided TBB is a safe diagnostic modality for peripheral lung lesions. Pneumothorax occurred in 3.3% (13/399) of procedures after EBUS-guided TBB and all these patients made a good recovery. Pulmonary emphysema and EBUS probe adjacent to the lesion were associated with a higher risk of post-procedure pneumothorax. To reduce the risk of pneumothorax, we suggest that the bronchoscopists introduce the probe into the bronchus, where it is imaged within the lung lesion, as far as possible.

## References

[1] Yoshikawa M, Sukoh N, Yamazaki K, Kanazawa K, Fukumoto S, et al (2007) Diagnostic value of endobronchial ultrasonography with a guide sheath for peripheral pulmonary lesions without X-ray fluoroscopy. Chest 131: 1788–1793. 17565021 10.1378/chest.06-2506

[2] Chung YH, Lie CH, Chao TY, Wang YH, Lin AS, et al (2007) Endobronchial ultrasonography with distance for peripheral pulmonary lesions. Respir Med 101: 738–745. 17015004 10.1016/j.rmed.2006.08.014

[3] Eberhardt R, Ernst A, Herth FJ (2009) Ultrasound-guided transbronchial biopsy of solitary pulmonary nodules less than 20 mm. Eur Respir J 34: 1284–1287. 19460785 10.1183/09031936.00166708

[4] Steinfort DP, Khor YH, Manser RL, Irving LB (2011) Radial probe endobronchial ultrasound for the diagnosis of peripheral lung cancer: systematic review and meta-analysis. Eur Respir J 37: 902–910. 20693253 10.1183/09031936.00075310

[5] Kurimoto N, Miyazawa T, Okimasa S, Maeda A, Oiwa H, et al (2004) Endobronchial ultrasonography using a guide sheath increases the ability to diagnose peripheral pulmonary lesions endoscopically. Chest 126: 959–965. 15364779 10.1378/chest.126.3.959

[6] Huang CT, Ho CC, Tsai YJ, Yu CJ, Yang PC (2009) Factors influencing visibility and diagnostic yield of transbronchial biopsy using endobronchial ultrasound in peripheral pulmonary lesions. Respirology 14: 859–864. 19703067 10.1111/j.1440-1843.2009.01585.x

[7] Eberhardt R, Anantham D, Ernst A, Feller-Kopman D, Herth F (2007) Multimodality bronchoscopic diagnosis of peripheral lung lesions: a randomized controlled trial. Am J Respir Crit Care Med 176: 36–41. 17379850 10.1164/rccm.200612-1866OC

[8] Herth FJ, Eberhardt R, Becker HD, Ernst A (2006) Endobronchial ultrasound-guided transbronchial lung biopsy in fluoroscopically invisible solitary pulmonary nodules: a prospective trial. Chest 129: 147–150. 16424425 10.1378/chest.129.1.147

[9] Herth FJ, Ernst A, Becker HD (2002) Endobronchial ultrasound-guided transbronchial lung biopsy in solitary pulmonary nodules and peripheral lesions. Eur Respir J 20: 972–974. 12412691 10.1183/09031936.02.00032001

[10] Kikuchi E, Yamazaki K, Sukoh N, Kikuchi J, Asahina H, et al (2004) Endobronchial ultrasonography with guide-sheath for peripheral pulmonary lesions. Eur Respir J 24: 533–537. 15459129 10.1183/09031936.04.00138603

[11] The definition of emphysema. Report of a National Heart, Lung, and Blood Institute, Division of Lung Diseases workshop. Am Rev Respir Dis 132: 182–185. 4014865 10.1164/arrd.1985.132.1.182

[12] Foster WL Jr, Gimenez EI, Roubidoux MA, Sherrier RH, Shannon RH, et al (1993) The emphysemas: radiologic-pathologic correlations. Radiographics 13: 311–328. 8460222 10.1148/radiographics.13.2.8460222

[13] Kazerooni EA, Lim FT, Mikhail A, Martinez FJ (1996) Risk of pneumothorax in CT-guided transthoracic needle aspiration biopsy of the lung. Radiology 198: 371–375. 8596834 10.1148/radiology.198.2.8596834

[14] Izbicki G, Shitrit D, Yarmolovsky A, Bendayan D, Miller G, et al (2006) Is routine chest radiography after transbronchial biopsy necessary?: A prospective study of 350 cases. Chest 129: 1561–1564. 16778275 10.1378/chest.129.6.1561

[15] Hiraki T, Mimura H, Gobara H, Shibamoto K, Inoue D, et al (2010) Incidence of and risk factors for pneumothorax and chest tube placement after CT fluoroscopy-guided percutaneous lung biopsy: retrospective analysis of the procedures conducted over a 9-year period. Am J Roentgenol 194: 809–814. 20173164 10.2214/AJR.09.3224

[16] Topal U, Ediz B (2003) Transthoracic needle biopsy: factors effecting risk of pneumothorax. Eur J Radiol 48: 263–267. 14652144 10.1016/s0720-048x(03)00058-5

[17] Ko JP, Shepard JO, Drucker EA, Aquino SL, Sharma A, et al (2001) Factors influencing pneumothorax rate at lung biopsy: are dwell time and angle of pleural puncture contributing factors? Radiology 218: 491–496. 11161167 10.1148/radiology.218.2.r01fe33491

[18] Kazerooni EA, Lim FT, Mikhail A, Martinez FJ (1996) Risk of pneumothorax in CT-guided transthoracic needle aspiration biopsy of the lung. Radiology 198: 371–375. 8596834 10.1148/radiology.198.2.8596834

[19] Yeow KM, Su IH, Pan KT, Tsay PK, Lui KW, et al (2004) Risk factors of pneumothorax and bleeding: multivariate analysis of 660 CT-guided coaxial cutting needle lung biopsies. Chest 126: 748–754. 15364752 10.1378/chest.126.3.748

[20] Trupka A, Waydhas C, Hallfeldt KK, Nast-Kolb D, Pfeifer KJ, et al (1997) Value of thoracic computed tomography in the first assessment of severely injured patients with blunt chest trauma: results of a prospective study. J Trauma 43: 405–411. 9314300 10.1097/00005373-199709000-00003

[21] Ouellette DR (2006) The safety of bronchoscopy in a pulmonary fellowship program. Chest 130: 1185–1190. 17035454 10.1378/chest.130.4.1185

